# Nuclear transport genes recurrently duplicate by means of RNA intermediates in *Drosophila* but not in other insects

**DOI:** 10.1186/s12864-021-08170-4

**Published:** 2021-12-05

**Authors:** Ayda Mirsalehi, Dragomira N. Markova, Mohammadmehdi Eslamieh, Esther Betrán

**Affiliations:** Department of Biology, The University of Texas at Arlington, Box 19498, Arlington, TX 76019 USA

**Keywords:** Nuclear transport, Recurrent gene duplication, Gene turnover, Genetic conflict, *Drosophila*

## Abstract

**Background:**

The nuclear transport machinery is involved in a well-known male meiotic drive system in *Drosophila*. Fast gene evolution and gene duplications have been major underlying mechanisms in the evolution of meiotic drive systems, and this might include some nuclear transport genes in *Drosophila*. So, using a comprehensive, detailed phylogenomic study, we examined 51 insect genomes for the duplication of the same nuclear transport genes.

**Results:**

We find that most of the nuclear transport duplications in *Drosophila* are of a few classes of nuclear transport genes, RNA mediated and fast evolving. We also retrieve many pseudogenes for the *Ran* gene. Some of the duplicates are relatively young and likely contributing to the turnover expected for genes under strong but changing selective pressures. These duplications are potentially revealing what features of nuclear transport are under selection. Unlike in flies, we find only a few duplications when we study the *Drosophila* duplicated nuclear transport genes in dipteran species outside of *Drosophila*, and none in other insects.

**Conclusions:**

These findings strengthen the hypothesis that nuclear transport gene duplicates in *Drosophila* evolve either as drivers or suppressors of meiotic drive systems or as other male-specific adaptations circumscribed to flies and involving a handful of nuclear transport functions.

**Supplementary Information:**

The online version contains supplementary material available at 10.1186/s12864-021-08170-4.

## Background

**Fig. 1:**
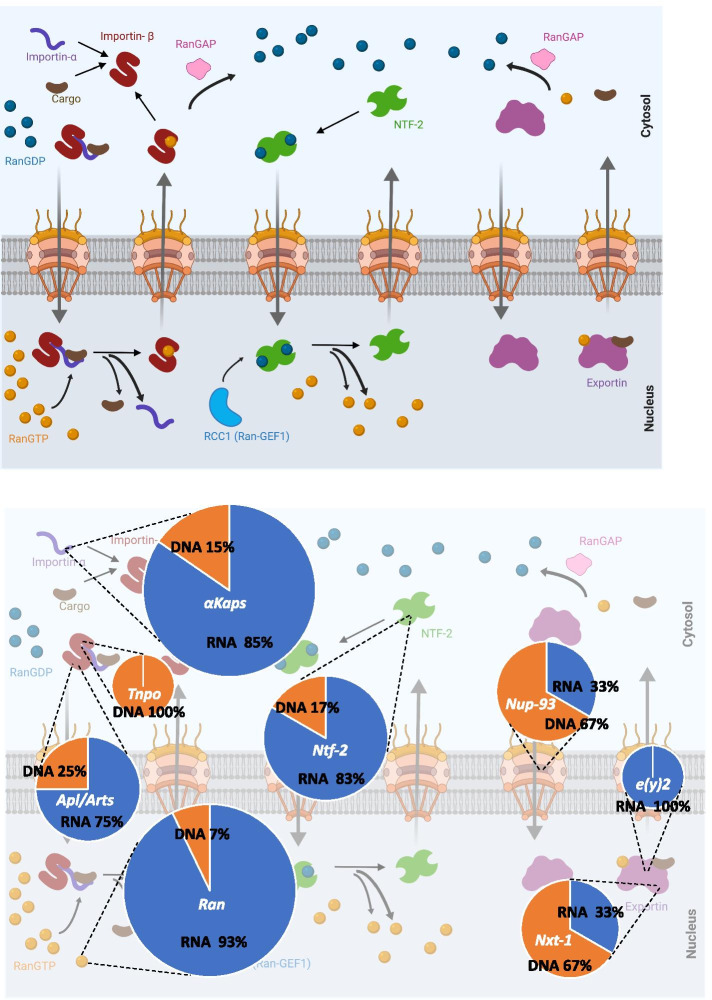
**A** Schematic view of conventional nuclear transport (Redrawn from [6, 7] with modifications). The nuclear pores drawn across the double membrane are formed by nucleoporins. **B** Percentages of RNA- vs. DNA-mediated duplications for each gene. Size of the pie charts are relative to the total number of independent duplication events for that gene

In eukaryotes, the nucleus is separated from the cytoplasm by a double membrane nuclear envelope. The nuclear envelope prevents the free flow of macromolecules between the nucleus and the cytoplasm. Selective nucleocytoplasmic transport of proteins and RNAs occurs through the nuclear transport system [1]. The conventional nuclear transport system consists of several components that fall into three main categories: 1) Nuclear pore complexes (NPCs) that are huge protein complexes residing on the nuclear envelope and consisting of several copies of approximately 30 different nucleoporins (Nups) that assemble to make the NPCs [2, 3]. 2) Nuclear transport receptors/carriers called karyopherins that consist of importins and exportins. The Importin superfamily consists of importin-α and importin-β sub-groups [4]. The carrier proteins recognize and translocate the cargo across the nuclear envelope through interactions with the NPCs. 3) Factors that assist the process and directionality of nuclear transport such as Ran, RCC1, RanGAP, and Ntf-2 [2, 5, 6]. These conventional mechanisms of receptor-mediated nuclear import and export in the case of proteins are depicted in Fig. 1A [6, 7]. Karyopherins are not only involved in protein import and export but are also involved in the transport of RNAs [6]. The type of cargo indicates which karyopherin will be used and if additional adaptors are needed [8]. Export of small RNAs such as tRNAs and microRNAs follow the general pattern of exportin-mediated protein export. In this case, tRNA/miRNA-specific exportins directly bind to tRNA/miRNA and RanGTP to mediate the export [8–10]. Export of large RNAs such as ribosomal RNAs (rRNAs) and mRNAs requires export receptors, as well as additional export adaptors assembled into complicated ribonucleoprotein (RNP) particles. The general mRNA export receptor complex in metazoans (Nxf1–Nxt1) is not a karyopherin family member [11]. Similar to the export of rRNAs and mRNAs, the export of snRNAs requires adaptor proteins that recruit the export receptor. However, assembly into an RNP is not needed for the nuclear export of snRNAs [8].

Aside from the conventional nuclear transport pathways, an increasing number of alternative non-conventional mechanisms have been described during the past years that mediate the nuclear transport independent of the karyopherins [12, 13]. These karyopherin-independent pathways include transport by alternative carriers such as the calcium-binding proteins calmodulin for nuclear entry and calreticulin for nuclear export. Also, some proteins can translocate through direct interaction with nucleoporins of the NPCs and be able to piggyback other cargoes with themselves across the nuclear membrane. Many proteins are transported by both conventional and alternative pathways. Exploiting multiple pathways for transport of the same cargo has been suggested to assure cellular functionality in the situations that one mechanism is inhibited or to adjust the nuclear transport based on cellular demand [12, 13].

Although nuclear transport mechanisms are quite conserved and needed in all eukaryotic cells [6], duplications of some of their components have been observed. One well characterized protein gene family is the importin family of adaptor proteins [14]. Karyopherin family members have a moderate sequence identity, and their highest similarity is for a binding domain for the small Ras-like GTPase Ran [5]. The diversity between karyopherins seems to have evolved in relation to their specific cargoes. However, although it was originally thought that each karyopherin can function as either import or export factor but not both, there is evidence that some karyopherins have import and export functions at the same time but for different cargo [15]. An N-terminal importin-β binding domain and a tandem array of Armadillo (ARM) repeats are characteristics of importin-αs, while importin-βs consist mainly of HEAT repeats. Previous studies have shown shared ancestry between ARM and HEAT repeats and importin-β being the progenitor of the importin-α karyopherins [16]. The importin family has diversified in higher eukaryotes. *Saccharomyces cerevisiae* encodes for a single importin-α, while most metazoans encode for three importin-αs [17]. However, this is not the case for most other components of nuclear transport, e.g., *Ran* and *Ntf2*, that are present as a single copy in most eukaryotic genomes.

Interestingly, previous studies have shown recurrent and convergent duplication of some of these single copy nuclear transport genes and expansions of the importin gene families in different *Drosophila* lineages. Losses of gene duplicates (i.e., high turnover), fast evolution, and particular patterns of gene evolution, and expression have also been observed in some *Drosophila* lineages [18–21].

Initially, *Ntf-2* and *Ran*, two nuclear transport genes, were reported to have given rise to retroposed copies at least three independent times within the same *Drosophila* lineages [18, 19, 21], but DNA mediated duplications were not explored. These duplicates have acquired testis-enriched expression and have shown signatures of positive selection in certain lineages [19, 21, 22]. Similarly, *importin-α* genes were later shown to have undergone six duplication events detected in the 12 initially sequenced *Drosophila* species genomes available in public databases. Some members of the *importin-α* gene family have been found to be repeatedly gained and lost with the retained copies gaining testis-enriched pattern of expression. On average, *Drosophila* genomes have been reported to contain between four to five importins [20].

Another member of the nuclear transport gene ontology (GO), *e(y)2b* was retroposed and generated *e(y)2*. This retroposition event is an example whereby a retrogene takes over the functions of the parental gene throughout evolution and gains ubiquitous expression while the parental gene, *e(y)2b* acquires a testis-enriched pattern of expression. E(y)2 was shown to be a component of a protein complex involved in transcription coupled mRNA export in *D. melanogaster* [23]. Another reported instance of gene duplication involving nuclear transport is the evolution of a pair of *D. melanogaster*-specific tandem duplicated genes, *Artemis* (*Arts*) and *Apollo* (*Apl*) from a 7.7 kb region on chromosome arm 3 L of *D. melanogaster*. The ancestral gene which has characteristics of *importin-β* was duplicated to produce two identical gene copies. However, after duplication, *Apl* and *Arts *evolved to acquire male-biased and female-biased expression, respectively. Investigations showed that* Apl *is male-biased and affects development and male fertility while* Arts *affects female fertility. These tissue-enriched patterns of expression might have evolved to resolve the sexually antagonistic effects of these genes [24].

Based on previous findings, mostly RNA-mediated duplication events contributed to the formation of new *importin-α*s, *Ntf-2, Ran* [18, 20], and *e(y)2* [23]. This recurrent duplication of *Ntf-2*, *Ran* and *importins* and the positive selection acting on some components of nuclear transport such as N*tf-2* and *Ran* duplicates*, RanGAP* and six nucleoporins (specifically *Nup107*) have been proposed to evolve in response to male germline conflicts, i.e., meiotic drive systems [19, 21, 25, 26]. Meiotic drive is a phenomenon in which heterozygous individuals favor transmission of one allele (selfish genetic element) to the gametes at the expense of the alternative allele and causes deviations from Mendel’s first law. Meiotic drive systems in *Drosophila* are often generated and evolve through gene duplication events [27].

Here, we present the results of a comprehensive study of nuclear transport duplications in insects. Nuclear transport genes (131 genes) were studied in 12 species of *Drosophila*. The duplicated genes in these species were studied in 10 additional *Drosophila* species and 29 species of non-*Drosophila* insects. These species encompass seven orders of insects representing divergence time of 583 Mya from *Drosophila* (Timetree.org). We explored if the same components of nuclear transport have been recurrently duplicated in *Drosophila* and in other insects. We also studied the duplication mechanisms and mode of evolution of these duplicates. Broad examination of the nuclear transport system duplications can reveal additional components that might have been under selection in testes, and if the selective pressures exist only in *Drosophila* or are broader, i.e., the same trends are observed in other insects. This study also reveals the contribution of RNA-mediated and DNA-mediated duplications to the observed patterns.

## Results

### Most nuclear transport duplicates are retrogenes in *Drosophila*

We analyzed duplications for 131 genes assigned to nuclear transport gene ontology (Supplementary material 1) initially in 12 *Drosophila* species genomes obtained from Ensembl Metazoa (species are listed in Supplementary material 2). In other *Drosophila* species (Supplementary material 2), we performed tBLASTn searches using the *Drosophila melanogaster* parental protein of each gene found to be duplicated in the initial search (Supplementary material 3). To find *importin-α-5* duplicates in *Drosophila*, we used *Drosophila eugracilis* parental protein as a query. We classify the duplications as retrotranspositions if the new gene is a complete retrocopy of the parental gene in which all coding exons are retained and is thus detected as a single hit in the Blast searches (See Methods). We analyzed the synteny across species for all recovered duplicated sequences to be certain of our classification as orthologous copies or independent duplications (Supplementary material 4). Our phylogenetic results support the synteny based conclusions (See below).

**Fig. 2:**
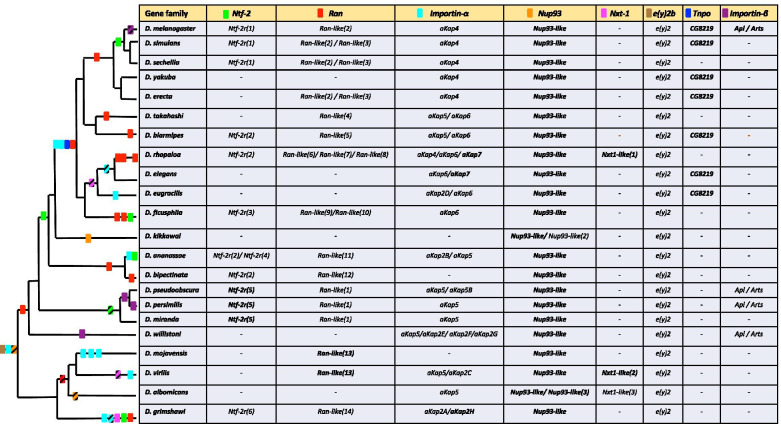
Presence and absence of gene duplicates across 22 species of *Drosophila* is shown. Summary of nuclear transport genes duplication events across the *Drosophila* species is shown in the phylogeny. Each rectangle represents a duplication event. Duplication events are shown at an approximate distance from the tips based on the percent identity to the parental gene protein in that species (Supplementary material 4). DNA-mediated duplications are shown in bold in the table and with striped boxes in the phylogeny

*Drosophila* duplication events detected for each gene are depicted in Fig. 2. Among the 131 nuclear transport genes that we explored, eight classes of genes were detected to have undergone duplication events: *Ran*, *Ntf-2*, *importin-α*s*, Nucleoporin-93* (*Nup93*), *NTF2-related export protein-1* (*Nxt-1*)*, enhancer of yellow 2* (*e(y)2), Transportin* (*Tnpo*) and *importin-β,* with *Ran, importin-α,* and *Ntf-2* being the three components experiencing the highest numbers within genomes (Figs. 1B and 2 and Supplementary material 4).

**Fig. 3:**
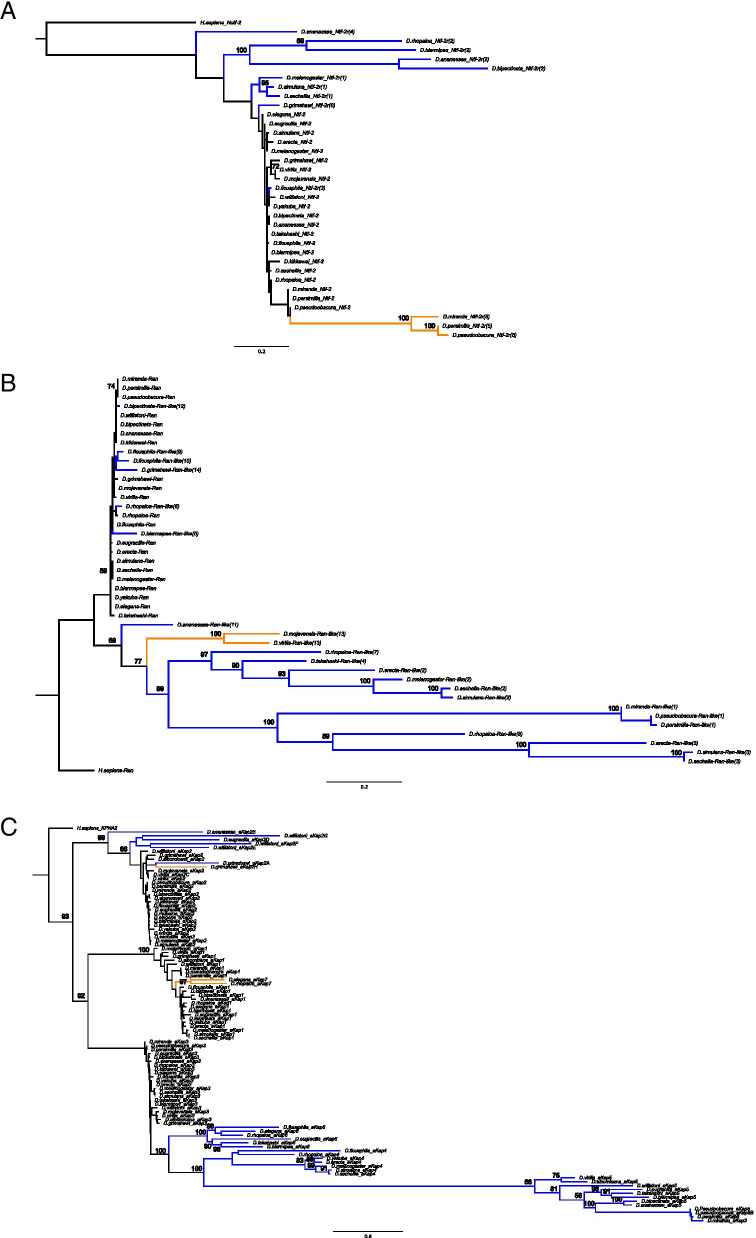
Maximum-likelihood tree constructed using PhyML showing the phylogenetic relationships between parental and duplicates of *Ntf-2* (**A**), *Ran* (**B**) and *importin-αs* (**C**) based on amino acid sequences. DNA mediated duplication are shown in orange and RNA-mediated duplications are shown in blue. Bootstrap values refer to 100 samples on PhyML performed using geneious software

Using *Drosophila melanogaster*’s Ntf-2 as a query we identified a total of six independent duplication events for *Ntf-2* in the *Drosophila* species examined. Three of these duplicates have not been reported before [18, 19] and include only one DNA-mediated duplication event (*Ntf-2r(5*)). All duplicates possess the characteristics of functional copies. Arbitrarily, we consider a copy to be a pseudogene if it has a premature stop codon that removes at least 10 amino acids. *Ntf-2* has four exons with four transcripts being annotated for this gene in FlyBase. All detected retroduplications are derived from *Ntf-2* transcript RA and the protein encoded by this transcript is used in the phylogeny in Fig. 3A. Transcript RA is the isoform that is expressed highest among all the transcripts [28, 29]. However, interestingly, the only detected DNA duplicate (*Ntf-2r(5*)) is not a tandem duplication and lacks the specific exon of Ntf-2 transcript B, indicating that this duplicate has lost this exon after duplication. *Ntf-2* duplicates through a DNA intermediate have not been previously reported for this gene. Understanding the functional difference between Ntf-2-RA and -RB might be relevant to understanding the selection acting on those duplicates.

Interestingly, *Ran* has been the gene with the highest turnover in terms of gain and loss events. By using *D. melanogaster*’s Ran as a query, we detected a total of 13 instances of independent duplication events of *Ran* with only one of these being a DNA-mediated duplication (Figs. 2 and 3B). Three of these duplication events have been reported in previous studies [18, 19]. *Ran* is a gene with two exons and only one transcript that has given rise to these duplicates. Unlike the *Ntf-2* duplicates that were all identified to be functional, at least 13 pseudogenes were identified for *Ran* in different *Drosophila* species (Supplementary material 4). Interestingly, only one of these pseudogenes is young (> 90% protein identity to the parental gene) and the rest are older. Six of those pseudogenes correspond to the loss of a functional copy, i.e., under strong purifying selection (See below), in that species lineage. Identification of several gain and loss events through gene duplication and pseudogenizations indicates a high turnover of these genes across *Drosophila* species. The high number of *Ran* pseudogenes (13/22 = 0.59 pseudogene per gene and per genome) is interesting and significantly higher (*P* < 0.0001 in Z-test) than the genome average, 0.0077 pseudogenes per gene per genome [30]. High number of detected pseudogenes is specific to *Ran* and we have not observed as many pseudogenes and turnover for other nuclear transport genes. It should be mentioned that the five *Ran* pseudogenes that are truncated (i.e., 10 to 38 amino acids short) remain to be experimentally verified for their potential functionality.

Using Importin-α1 as a query, we identified a new *importin-α* gene which we named as *αKap7* (*α-Karyopherin-7*) following the nomenclature from previous work [20] for consistency (Fig. 2 and Supplementary material 4). αKap7 possess both an N-terminal IBB domain as well as Armadillo repeats which are the characteristics of the Importin-αs. *αKap7*, which is present in *D. rhopaloa* and *D. elegans*, is evolutionary closely related to *αKap1,* confirmed with both identity scores from Blast searches and phylogenetic relatedness (Fig. 3C and Supplementary material 3). We have also detected one copy of *αKap7* in *D. willistoni* which has been pseudogenized (Supplementary material 4).

Blast searches with *importin-α2* revealed that this gene has given rise to several (8) independent duplications. Three independent duplications from *αKap2* (*αKap2A*, *αKap2B* and *αKap2C*) were reported before [20]. We found five more independent duplications from *αKap2* in the Blast searches of 22 *Drosophila* genomes (Supplementary material 4). We identified a new *αKap2* duplicate in *D. eugracilis* (*αKap2D*), three independent duplicates in *D. willistoni* (*αKap2E*, *αKap2F* and *αKap2G*) and an additional duplicate in *D. grimshawi* (*αKap2H*) (Fig. 2). We follow previous nomenclature [20] to indicate that they are duplicates from *αKap2*. All of these newly detected *αKap2* duplicates contain the Armadillo repeats, characteristic of the canonical *importin-α*s and they all lack the IBB domain at their N-terminal. *αKap2D*, *αKap2E*, *αKap2F* and *αKap2G* are retrogenes as they do not contain any of the introns of *αKap2*. *αKap2H* is a partially processed retroduplicate and contains one of the introns of the *αKap2* gene.

Using Importin-α3 as a query, we identified presence of *αKap4* in two species outside of the *melanogaster* subgroup (Fig. 2). Previously the presence of *αKap4* was reported to be only in *melanogaster* species subgroup [20]. These αKap4s lack the IBB domain and contain one of the introns of *αKap3* as described before for this importin.

Exploring *Drosophila* species with αKap3 duplicates we identified another independent duplication event which we named as *αKap6*. This duplicate that is present in six *Drosophila* species (Fig. 2 and Supplementary materials 3 and 4), also lacks the IBB domain at the N-terminus and appears to be a partially processed retroduplicate by having a similar structure to *αKap4*s and containing only one intron from the parental sequence.

We used *D. eugracilis* αKap5 to identify further duplications of this gene. No new independent duplications were found for *αKap5*. However, we detected the presence of *αKap5* in four species that have not been explored in the previous studies, two of these species are close to the *D. melanogaster* group. We have found that *αKap5* has been pseudogenized in *D. mojavensis* as it possesses several premature stop codons.

Finding additional species containing *αKap4* and *αKap5* provides a more precise dating of *αKap5* loss and origination of *αKap4* than previously reported.

Interestingly, the results from Importin duplications shows that the majority of independent duplication events occurred for *importin-α2*, one importin that was characterized in previous studies to have specialized in *Drosophila* for gametogenesis functions [17, 20, 31, 32].

Our conserved domain analysis of newly detected *αKap4s*, *αKap5s, αKap6* and every new duplication of *αKap2* (*αKap2D-2H)* shows absence of the IBB domain in these duplicates. Similarly, Phadnis et al. showed absence of IBB domain in *αKap4, αKap5 and αKap2A,* highlighting the presence of at least one testis-enriched *importin-α* that lacks the IBB domain in all 22 *Drosophila* species that have been studied with the exception of *D. kikkawai*. IBB-less *importin-αs* have been shown to be functional in nuclear transport in *S. cerevisiae* and *Drosophila* [33, 34]. Origination of several *importin-αs* that lack the IBB domain might be an adaptation for *Importin-β* independent and specialized nuclear transport function in the male germline.

Among other nuclear transport-related genes examined here for *Drosophila*, there are few others that have undergone duplication events. Of all 30 nucleoporins we examined, *Nup93* is the only nucleoporin that has been duplicated. We have found an old DNA duplication event which is present in all 22 *Drosophila* species we analyzed. In addition, two other independent duplicates were found. One retroduplicate present is *D. kikkawai* and a second DNA-mediated duplicate with the least identity to its parental gene in *D. albomicans* (Supplementary materials 4 and 5A). We could not find any traces of pseudogenization in these duplicates and despite being relatively old, all detected *Nup93* duplicates seem to be functional.

*Nxt-1* is another nuclear transport component found to have undergone duplications. We discovered three independent duplications for this gene and all copies seem to be functional. Two out of the three duplication events of *Nxt-1* are DNA-mediated but not in tandem (Fig. 2 and Supplementary materials 4 and 5B).

*Tnpo* (*βKap2*) is another gene that has a duplication present in seven species of *Drosophila*. The shared synteny shows that all these duplicates are orthologous and derive from a single DNA-mediated duplication event (Fig. 2 and Supplementary material 4, and 5C).

An additional detected gene family of nuclear transports is *e(y)2b* which has retroposed and generated *e(y)2*. This previously described retroposition event is an exceptional example in which the retrogene (*e(y)2*) takes over the functions of the parental gene (*e(y)2b*) during evolution and gains ubiquitous expression while the parental gene has acquired testis-enriched pattern of expression [23]. In the previous study of this gene family, [23] presence of *e(y)2* and *e(y)2b* in eight species of *Drosophila* was reported. Our analysis shows the existence of both parental and duplicate genes in the 22 *Drosophila* species studied here indicative of one old retroduplication event (Fig. 2 and Supplementary materials 3, 4 and 5D). All the retrieved sequences seem to be functional.

Our results show a greater number of duplication events and losses for nuclear transport genes in *Drosophila* than was previously reported. This study has also revealed an excess of RNA-mediated duplications (Fig. 1B) that cannot be explained by a lower detection probability of DNA duplicates because using single exons as queries for our Blast searches showed the same results (See Methods).

### The high turnover of nuclear transport genes is circumscribed to *Drosophila*

We explored duplication of the nuclear transport components duplicated in *Drosophila* in 29 non-*Drosophila* insect species representing seven orders of insects. For these searches outside of *Drosophila*, we used each species’ specific parental protein as a query. Despite extensive detection of gene duplications for these genes in *Drosophila* using this approach, no functional gene duplicate for those genes was found in species outside of Diptera. Our analysis shows that nuclear transport duplication events are not only limited to *Drosophila* lineages. However, the duplication events are observed in fewer genes and are limited to three dipteran species close to *Drosophila* and not any species outside of Diptera contain this kind of functional nuclear transport duplicates (Supplementary materials 3 and 4). Dipteran duplicates detected out of *Drosophila* are limited to *Glossina morsitans morsitans* (Tsetse fly), *Aedes aegypti* (Yellow fever mosquito) and *Anopheles gambiae* (Malaria mosquito)*.* We observed retroduplicates of *Ntf-2* in *Glossina morsitans morsitans* and *Anopheles gambiae*. *Glossina morsitans morsitans Ntf-2* duplicate has been pseudogenized. Similarly, *Ran* has given rise to duplicates outside of *Drosophila* species. We observed retroduplicates of *Ran* in *Glossina morsitans morsitans* and *Aedes aegypti*. *Aedes aegypti* duplicate has been pseudogenized*. Nxt-1* has also one retroduplicate in *Aedes aegypti*, and *Nup93* has a retroduplicate in *Glossina morsitans morsitans.* Our analyses show presence of *importin-α1*, *importin-α2* and *importin-α3* in all 29 outside *Drosophila* insect species, however unlike *Drosophila*, we did not observe any additional *importin-α* duplicates in non-*Drosophila* species. Likewise, no duplication of *e(y)2b* and *Tnpo (βKap2)* is detected in non-*Drosophila* species (Supplementary materials 3 and 4).

Scarcity of duplicates out of *Drosophila* species cannot be explained by the quality of the genomes (Supplementary material 2) or the divergence of the duplicates. For example, if we focus on duplicates with > 80% identity to their parents per species, *Drosophila* has a higher proportion of *Ran* duplicates than in other insects genomes (i.e., 30% (7/22) in *Drosophila* and only 3% (1/29) in other insects including Diptera). These two proportions were tested with a Z-test. (Z = 2.7591; *P* = 0.0058). Likewise, average number of *Ntf-2* duplicates with > 80% identity to their parents per species is also significantly higher in *Drosophila* than in other insects (i.e., 23% (5/22) in *Drosophila* and only 3% (1/29) in other insects; Z = 2.1164; *P* 0.034).

### Nuclear transport gene duplicates in flies have testis-enriched expression

Expression data from *D. melanogaster* modENCODE RNA-Seq data for different tissues [28, 29] (Supplementary material 6) and several previous studies in additional *Drosophila* species showed that nuclear transport genes follow a general pattern in which the ancestral genes actively express in almost every tissue, while the duplicate genes evolve to have a tissue-enriched expression in testis. RT-PCR results from *D. melanogaster* and *D. ananassae* showed that *Ntf-2r* and *Ran-like* are strongly testis enriched [19, 21, 35], while the parental genes, *Ntf-2* and *Ran* are expressed in every tissue with a higher expression in the ovaries.

Profiled expression pattern studied by RT-PCR analyses from RNA collected from various species of adult *Drosophila* male and female tissues showed that *aKap4*, *aKap2B*, *aKap2C*, *aKap5A* and *aKap5B* have gained a highly testis-enriched pattern of expression in contrast to the *aKap1* and *aKap3* that have a ubiquitous expression and *aKap2* which is enriched in both testes and ovaries [20]. This is in agreement with published profiles of gene expression for those genes [35].

*Tnpo*, the ancestral gene of *CG8219* has ubiquitous expression with high expression in ovary and the duplicate gene has acquired high testis-enriched expression. Similarly, while the expression of *e(y)2* was detected in all tissues at the same level, the mRNA of *e(y)2b* was detected only in testis [23]. modENCODE RNA-Seq for *D. melanogaster* data shows very low to moderate expression of *Nup93* (*CG11092*) in every tissue and high expression of this gene in the ovary, while *Nup93-like* (*CG7262*) has very low to moderate expression in other tissues but acquired moderately high expression in testis.

Therefore, accumulated expression data available for the duplicated genes and parental genes supports presence of testis-enriched expression pattern for all duplicated genes for which expression has been studied.

### Mode of evolution and phylogenetic analyses of the duplicates

To examine the evolutionary relationships between parental genes and duplicates, we performed phylogenetic analysis using the protein sequences and a maximum likelihood (ML) approach in 22 *Drosophila* species. We found that parental and duplicate genes are grouped into distinct clades in which parental sequences are associated with short branch lengths consistent with a slower rate of evolution for parental sequences than duplicates suggesting a high degree of evolutionary constraints for parental genes. In contrast, duplicate genes have long branches indicating that they are evolving at a significantly faster rate than their respective parental counterparts (See below).

To describe the selective pressures acting on parental and duplicated genes newly discovered here, first, the ratio of nonsynonymous substitutions per nonsynonymous sites to synonymous substitutions per synonymous sites (dN/dS or *ω*) was calculated using the CodeML algorithm (Yang, 2007) in EasyCodeML (www.github.io/bioeasy/EasyCodeML; Supplementary material 6). The dN/dS ratio was smaller than 1 for all genes tested, indicating that purifying selection is the major evolutionary force at the protein level (Table 1). However, the mean *ω* values for the parental genes in the species compared (*ω*_*Ran(1)*_ = 0.0001, *ω*_*Ran(3)*_ = 0.0147, *ω*_*Ntf-2(5)*_ = 0.0345, *ω*_*Nup93*_ = 0.1281, *ω*_*αKap3*_ = 0.0224) were lower than the *ω* values for the retroduplicates, (*ω*_*Ran-like(1)*_ = 0.47031, *ω*_*Ran-like(3)*_ = 0.5177, *ω*_*Ntf-2r(5)*_ = 999, *ω*_*αKap6*_ = 0.354) (Supplementary material 6) except for Nup93-like (*ω*_*Nup93-like(1)*_ = 0.0827). All ratios were statistically significantly smaller than 1 except for *Ran-like(1)* (Supplementary material 6). In *Ran-like(1)*, we see the duplicate only in three very close related species (i.e., where few synonymous changes have occurred) and that might render this estimate less reliable.

**Table 1:**
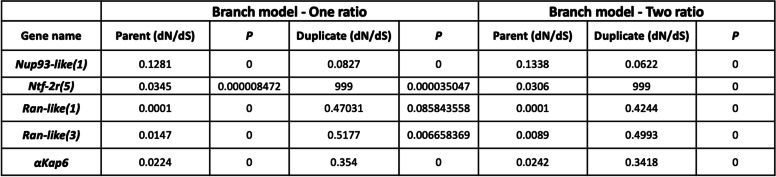
Ratio of nonsynonymous substitutions per nonsynonymous sites to synonymous substitutions per synonymous sites (dN/dS; *ω*) for selected parental and newly described gene duplicates

One-ratio model was used to test if the *d*N/*d*S ratio was significantly different from a ratio of one. Two-ratio branch model was used to test whether parental gene and new gene evolve at different rates. The *P*-value is shown for these likelihood ratio tests. See Methods and Supplementary material 6 for all details

Two-ratio branch model can be used to test whether there are significant *ω* differences among branches of the tree by assuming that specific branches can have an *ω* that differs from the rest of the tree [36–39]. So, we performed two-ratio branch model analysis further confirming that parental sequences are subjected to highly significantly stronger purifying selection than the duplicates (*p* < 0.001) (Table 1 and Supplementary material 6), with the exception of *Nup93* in which a higher rate is observed for the parent instead of the duplicate. This is in agreement with our interpretation of the branch lengths observed from the phylogenetic analysis of each set of genes (Fig. 3A-C, and Supplementary material 5A-D).

Previous studies showed that novel *αKaps* (*αKap2B* and *αKap5)* evolve under purifying selection except for ARM repeats of *αKap4* which showed to be evolving under positive selection [20]. Also, while both *Ntf-2* and *Ran* were shown to be evolving under purifying selection in *Drosophila* with *Ran* being under stronger purifying selection (dN/dS = 0.0188 and 0.0065 respectively), *Ran-like* and *Ntf-2r* were shown to be evolving under both purifying and positive selection in certain *Drosophila* lineages [19, 21]. Therefore, the statistically significant higher rate of evolution for the duplicated genes is at least partially explained by positive selection as supported by the McDonald and Kreitman tests and site specific likelihood models [19–21].

## Discussion

The majority of new gene duplicates (∼80%) that are limited to single species are tandem gene duplications [40] and CNVs are often DNA mediated duplicates [41] in *Drosophila* revealing the mutational biases in these genomes. In contrast, in this study we confirm and expand on the finding that the vast majority of nuclear transport duplications in *Drosophila* are relocations (copies to a different location than the parental gene) (Supplementary material 3), fast evolving and many are RNA-mediated (78%). These new genes are, however, under strong purifying selection and/or positive selection supporting their functional relevance. We interpret the mode of evolution of these nuclear transport-derived gene duplicates to be related to the function retroposed copies are selected for in male germline. This is the way these patterns have been interpreted before. Relocation or retroposition might allow these copies to acquire testis-enriched expression and the lack of introns might be beneficial for the processing and nuclear export of the transcripts during meiosis in male germline [42–44].

Although we find a few duplications of nuclear transport genes in dipteran species outside of *Drosophila*, most of them are in *Drosophila* species and duplicates for those genes are not found in other insect genomes. Total number of duplicate genes per species is shown in Supplementary material 6 indicating presence of at least 3 gene duplicates in *Drosophila* species. The species with the highest number of nuclear transport duplicates is *D. rhopaloa* (10 gene duplicates), while *Glossina morsitans morsitans,* with only two gene duplicates, has the highest number of nuclear transport duplicates among other dipteran species. The other two species, *Aedes aegypti* and *Anopheles gambiae* have only one gene duplicate.

The number of independent duplication events per gene (Fig. 2, Supplementary materials 3 and 4) shows greatest number of duplications for *Ran*, i*mportin-α* and *Ntf-2-RA* (with 13, 13 and 6 duplication events, respectively) and in particular i*mportin-α2* (8 duplication events). These few gene families of nuclear transport with a high number of gene duplications reveal what features of nuclear transport might be under selection in *Drosophila*. These are all genes or transcripts that are highly or specifically expressed in *Drosophila* testis. Significant number of *Ran*, *importin-α* and *Ntf-2* duplications could be an adaptation for the suppression of male meiotic drive systems similar to Segregation Distortion (SD) of *D. melanogaster*. The turnover observed for *Ran* and *importin-α* occurs for functional genes (*Ran-like(2)*, *Ran-like(3)*, *Ran-like(10)* and *importin-α5*) that are becoming pseudogenes or being lost in some lineages (This work [19–21]) speaks of strong but changing selective pressures consistent with this view.

Segregation Distorter (*SD*) as one of the well-characterized meiotic drive systems described in *D. melanogaster* is a multi-locus gene complex comprised of three main interacting loci clustered near the centromere of chromosome 2. The three loci are, 1) The driver, segregation distorter (*Sd*), which is the main distorting locus and a truncated tandem duplication of the *RanGAP* gene, 2) Enhancer of *SD* (*E(SD*)), and 3) The target of drive, *Responder* (*Rsp*), which is a large array of 120 bp pericentromeric satellite repeats. The copy number of these repeats define the sensitivity of the *Rsp*. The *Rsp* alleles with < 200 copies are insensitive, and those alleles with > 700 copies are sensitive. Molecular characterization of the *SD* components showed that *SD* chromosomes carry insensitive alleles of *Rsp* (*Rsp*^*i*^), while the wildtype alleles mainly carry the sensitive alleles (*Rsp*^*s*^). With the three defined loci, the genotypes of the *SD* and wildtype chromosomes will be *Sd E(SD) Rsp*^*i*^ and *Sd*^*+*^*E(SD)*^*+*^ *Rsp*^*s*^ respectively. *SD* causes meiotic drive in which although heterozygous *SD/SD*^+^ females pass *SD* and *SD*^*+*^ equally to their progenies, 99% of heterozygous *SD*/*SD*^*+*^ male progenies inherit *SD* bearing chromosomes. *Sd* performs the same enzymatic activity as the wild-type *RanGAP*. However, since it is truncated it lacks its nuclear export signal (NES), and it mislocalizes to the nucleus resulting in accumulation of RanGDP molecules in the nucleus [25]. Disruption of the RanGTP gradient might cause segregation distortion. The reason why only *SD*-bearing sperms survive spermatogenesis might be the disruption of chromatin remodeling in *SD*^+^-bearing spermatid nuclei and the failure of the transition between histones and protamines and maturation into functional sperm, resulting in their accumulation in the waste bags [45].

Recent studies have suggested the involvement of Piwi-interacting RNA (piRNA) pathway in silencing the *Rsp* satellite repeats needed for maturation of normal spermatids.

**Fig. 4:**
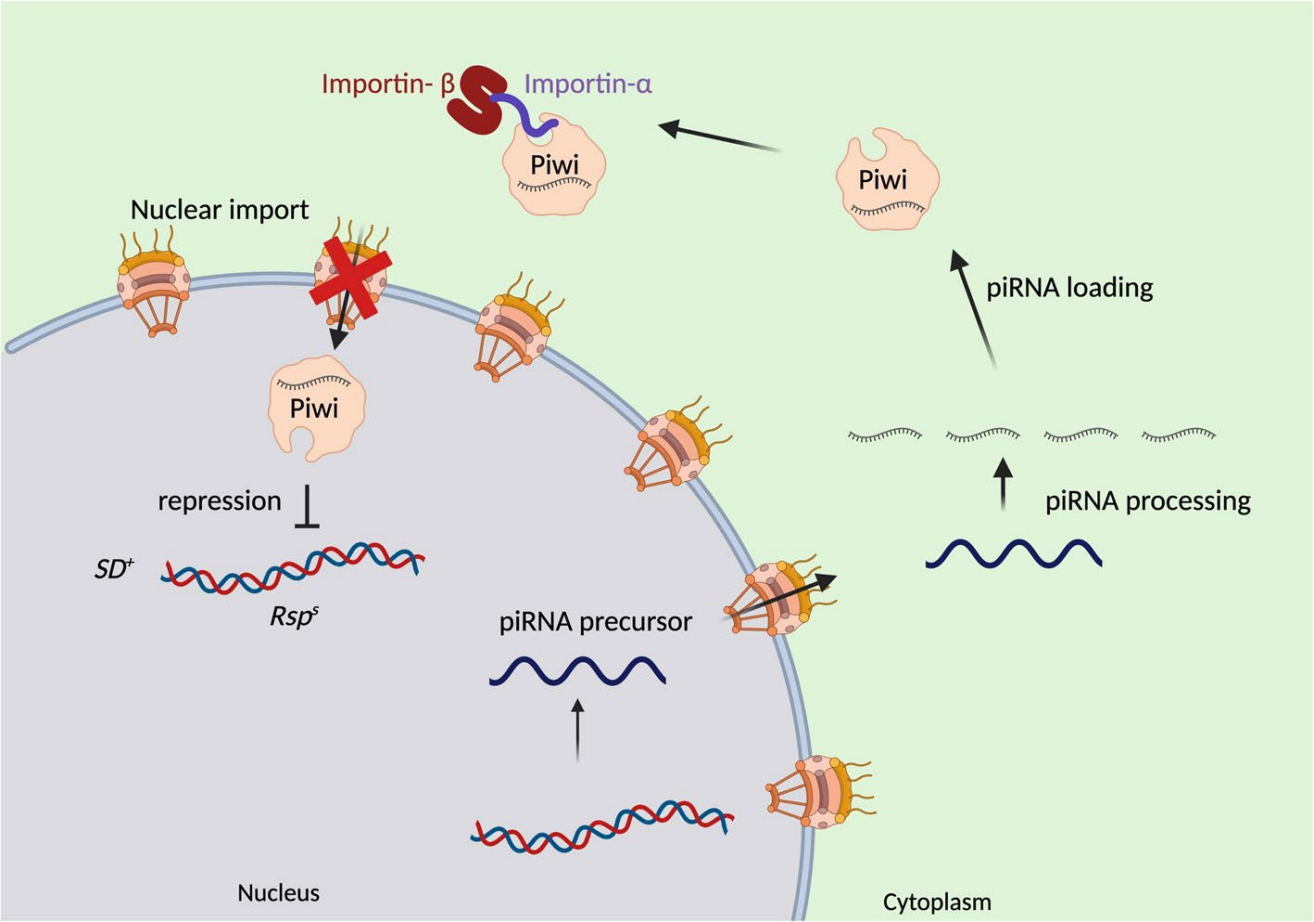
Proposed model of how the piRNA pathway might be involved in the SD system in *Drosophila*. piRNA precursors are exported from the nucleus to the cytoplasm, where piRNA biogenesis and loading of the RNA to the Piwi protein happens. Import of this complex to the nucleus has been proposed to be needed for chromatin condensation of the *Rsp* repeats and in SD it might not occur due to disruption of Ran-GTP/Ran-GDP gradient [28, 33, 34]

The piRNA pathway is a germline specific small RNA-based silencing system. In *Drosophila*, most of piRNAs derive from piRNA clusters that are large loci with high proportion of TE sequences [46, 47]. piRNA precursors which are piRNA cluster transcripts are exported from the nucleus. In the cytoplasm several factors contribute to the piRNA biogenesis and loading of piRNA into Piwi proteins. After formation of piRNA-induced silencing complexes (pi-RISCs), they are imported back into the nucleus, where they guide transcriptional silencing of repetitive elements by inducing heterochromatin formation at the target loci (Fig. 4) [48]. Piwi is the nuclear member of the Argonaute protein family in *Drosophila*. The two other family members, Aubergine (Aub) and Argonaut3 (AGO3), remain in the cytoplasm and function in the ping-pong cycle. These proteins, Piwi, Aubergine (Aub), and Argonaut3 (AGO3), are specifically expressed in germline, and maintain the integrity of the genome during gametogenesis by silencing of the transposons and other repetitive elements [47]. The need of proper import and export mechanisms for the functionality of piRNA pathway in silencing repetitive elements highlights the importance of nuclear transport components in suppressing meiotic drive systems. Previous studies in *Drosophila* ovaries have shown that *importin-α* have a crucial role in the localization of Piwi to the nucleus. In the loss of function mutants of *importin-α*2 and *importin-α*3, Piwi is not imported into the nucleus, but this can be rescued by overexpression of any of the Importin*-α* member [49]. Investigations of whether the *Responder* array of satellite repeats could be a target of the piRNA pathway has shown that mutations to both *Aubergine* and *Piwi* act as enhancers of distortion [47].

In the presence of *SD/SD*^+^ males which may suffer disruption of the RanGTP gradient, the canonical import of piRNAs loaded into Piwi proteins may be perturbed. Thus, the piRNAs cannot efficiently be imported back to the nucleus for transcriptional silencing of the *Rsp* repeats (Fig. 4). In this process spermatids with *Rsp*^*S*^ alleles which have higher copy number of repeats than *Rsp*^*i*^ disproportionally get affected and fail to mature to normal spermatids [45]. Two other genes that show duplications (*Nxt-1* and *Nup93*) have been described to be part of the piRNA pathway in ovaries [48]. The positive selection observed for some of these duplicates [19–21] or the effects of the extra dose of genes like *Ran* [50] on the suppression of SD would support this. Alternatively, the many duplicates of *Ntf-2*, *Ran* and *importin-α* could have other male-specific functions. In any case, the presence of these duplicates mostly in *Drosophila* would support that these conflicts or other testis-enriched selective pressures are restricted to fruit flies.

## Methods

### Identification of orthologous and paralogous genes of nuclear transport components in 12 *Drosophila* species

We analyzed genes assigned to Gene Ontology ID, GO: 0051169 (Nuclear transport; 131 genes) to detect the duplicates of nuclear transport components. BlastP searches [51] considering a cutoff level of > = 50% identity were performed for 12 *Drosophila* species genomes obtained from Ensembl Metazoa for every protein included in the above Gene Ontology. Using the Markov Cluster Algorithm (MCL Algorithm) [52], genes were grouped into gene families. Gene families with more than one member were considered to have duplications and were manually analyzed in order to understand their detailed structure. We used previously published scripts for these analyses [53].

### Identification of analogous duplications of nuclear transport components in additional insect lineages

We expanded our search of the candidate nuclear transport genes’ duplications in more *Drosophila* species as well as outside of *Drosophila* to see if the same selective pressures exist in all analyzed species. Searches specifically for the duplication of *Ntf-2*, *Ran*, *importin-α****,*** *Nup93*, *Nxt-1*, *e(y)2b*, *Tnpo* and *importin-β* were performed for a total of 22 *Drosophila* species and 29 non-*Drosophila* insect species listed in Supplementary material 1; for which full genome sequences are available on FlyBase. We used tBLASTn implemented in FlyBase [54] or NCBI genome databases. *Drosophila melanogaster* parental protein of each gene was used as a query for searches within the *Drosophila* genus. For finding additional *importin-α5* duplicates we used *Drosophila eugracilis* as a query. For searches outside of *Drosophila*, we used each species’ specific parental protein as a query. tBLASTn hits with identity > = 40% were retained. We analyzed all tBLASTn results to understand the mode of evolution of the duplicated gene copies. In cases of retroduplication, the gene copy appears in BLAST searches as a solid hit with no introns in between. While in a DNA-mediated duplication event, the exons appear as individual hits on the same chromosome or scaffold. In order not to miss a DNA duplication event, we also used single exons as queries of our Blast searches, which did not change the results. For annotated genomes, we used the FlyBase genome browser track option to obtain all sequences. For genomes that were sequenced but not annotated, we used the Graphics track option of tBLASTn in the NCBI genome database to collect the complete sequence of the hits. We analyzed and annotated all gathered parental and duplicate sequences manually using the ExPASy bioinformatics resource portal [55], thus allowing us to confirm their functionality and detect pseudogenes. If one or more premature stop codons were observed in the translated proteins or the transcript was truncated for at least 10 amino acids, the copy was considered a pseudogene. The results of all BLAST searches can be found in Supplementary material 3.

Using the NCBI conserved domain database [56, 57], we compared the regions flanking each duplicate for synteny conservation, thus allowing us to confirm if detected sequences are orthologs or independent duplications.

### Phylogenetic analyses

To examine the evolutionary relationships between parental genes and duplicates, we built phylogenetic trees using the maximum likelihood (ML) approach [58]. Multiple alignments of protein sequences were performed using ClustalW [59] implemented in Geneious software (Version 2020.1) [60]. Maximum likelihood phylogenetic trees of *Ntf-2*, *Ran*, *importin-α, Nup93, Nxt-1, e(y)2b* and *Tnpo* parental and duplicate sequences among 22 *Drosophila* species were constructed by using the BLOSUM62 substitution model with 100 bootstrap branch support in PhyML [61] applied in the Geneious software. We used FigTree (Version 1.4.4) (http://tree.bio.ed.ac.uk/software/figtree) and Inkscape software to modify visual features of the phylogenies. All protein sequences used are provided as Supplementary material 7.

### Mode of evolution analyses

To detect the signatures of selection on different genes, the ratio of nonsynonymous substitutions per nonsynonymous sites to synonymous substitutions per synonymous sites (*d*N/*d*S) was estimated and compared between newly described parental and new gene pairs using the CodeML algorithm (Supplementary material 6) [58] implemented in EasyCodeML [39]. Accordingly, under the assumption of neutral evolution, *d*N/*d*S ratios are expected to have a value of 1, ratios less than 1 indicate negative or purifying selection, corresponding to high selective constraints, and values greater than 1 indicate positive selection, suggesting adaptive evolution [62]. First, the branch model was used with a null model assuming that each respective group of sequences is evolving at the same rate (one-ratio model) and an alternative model in which the *d*N/*d*S ratio was fixed to 1. Second, to test whether the parental and the retroduplicate genes evolve under different evolutionary constraints, additional branch model specifying different rates for the different gene branches were compared to a single rate. The likelihood ratio test (LRT [63];) was conducted to perform pairwise comparisons of both models for all comparisons. Only a *P*-value of 0.05 or less in the LRTs was considered to be significant, indicating that the rates between parental gene and duplicate genes were significantly different (Supplementary material 6).

## Supplementary Information

**Additional file 1.**

**Additional file 2.**

**Additional file 3.**

**Additional file 4.**

**Additional file 5.**

**Additional file 6.**

**Additional file 7.**

## Data Availability

All sequences extracted from the genomes or analyzed during this study are provided in Supplementary material 6.
