# Supplementary material for: Nuclear transport genes recurrently duplicate by means of RNA intermediates in Drosophila but not in other insects
Source: BMC Genomics. 2021 Dec 5;22:876. doi: 10.1186/s12864-021-08170-4 (PMC8645118; doi:10.1186/s12864-021-08170-4)
Supplement: Supplementary file 5 — Additional file 5. [file 12864_2021_8170_MOESM5_ESM.pdf]

A.

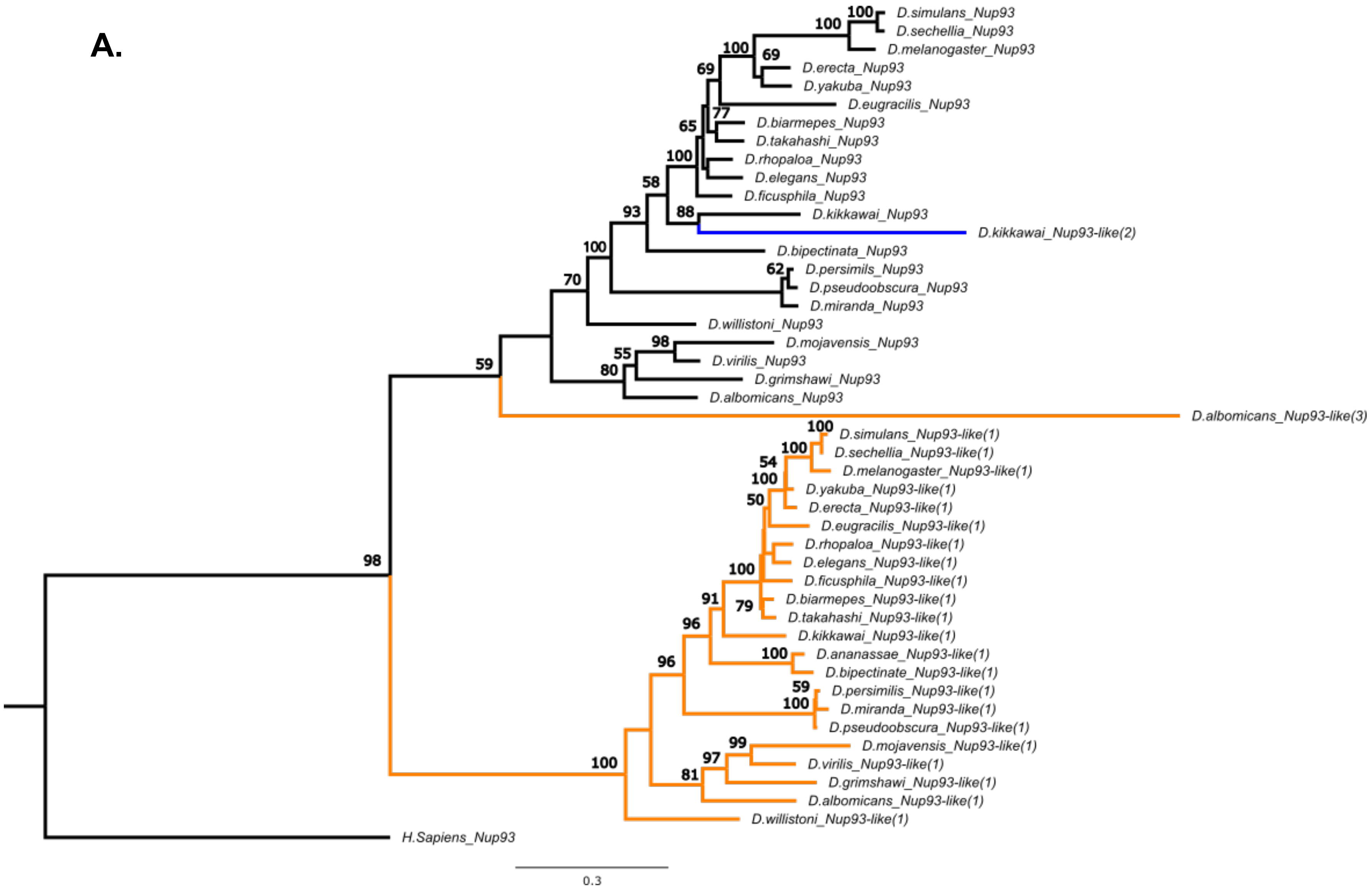
D.simulans_Nup93
D.sechellia_Nup93
D.melanogaster_Nup93
D.erecta_Nup93
D.yakuba_Nup93
D.eugracilis_Nup93
D.biarmepes_Nup93
D.takahashi_Nup93
D.rhopaloa_Nup93
D.elegans_Nup93
D.ficusphila_Nup93
D.kikkawai_Nup93
D.kikkawai_Nup93-like(2)
D.bipectinata_Nup93
D.persimils_Nup93
D.pseudoobscura_Nup93
D.miranda_Nup93
D.willistoni_Nup93
D.mojavensis_Nup93
D.virilis_Nup93
D.grimshawi_Nup93
D.albomicans_Nup93
D.albomicans_Nup93-like(3)
D.simulans_Nup93-like(1)
D.sechellia_Nup93-like(1)
D.melanogaster_Nup93-like(1)
D.yakuba_Nup93-like(1)
D.erecta_Nup93-like(1)
D.eugracilis_Nup93-like(1)
D.rhopaloa_Nup93-like(1)
D.elegans_Nup93-like(1)
D.ficusphila_Nup93-like(1)
D.biarmepes_Nup93-like(1)
D.takahashi_Nup93-like(1)
D.kikkawai_Nup93-like(1)
D.ananassae_Nup93-like(1)
D.bipectinate_Nup93-like(1)
D.persimilis_Nup93-like(1)
D.miranda_Nup93-like(1)
D.pseudoobscura_Nup93-like(1)
D.mojavensis_Nup93-like(1)
D.virilis_Nup93-like(1)
D.grimshawi_Nup93-like(1)
D.albomicans_Nup93-like(1)
D.willistoni_Nup93-like(1)
H.Sapiens_Nup93
0.3

B.

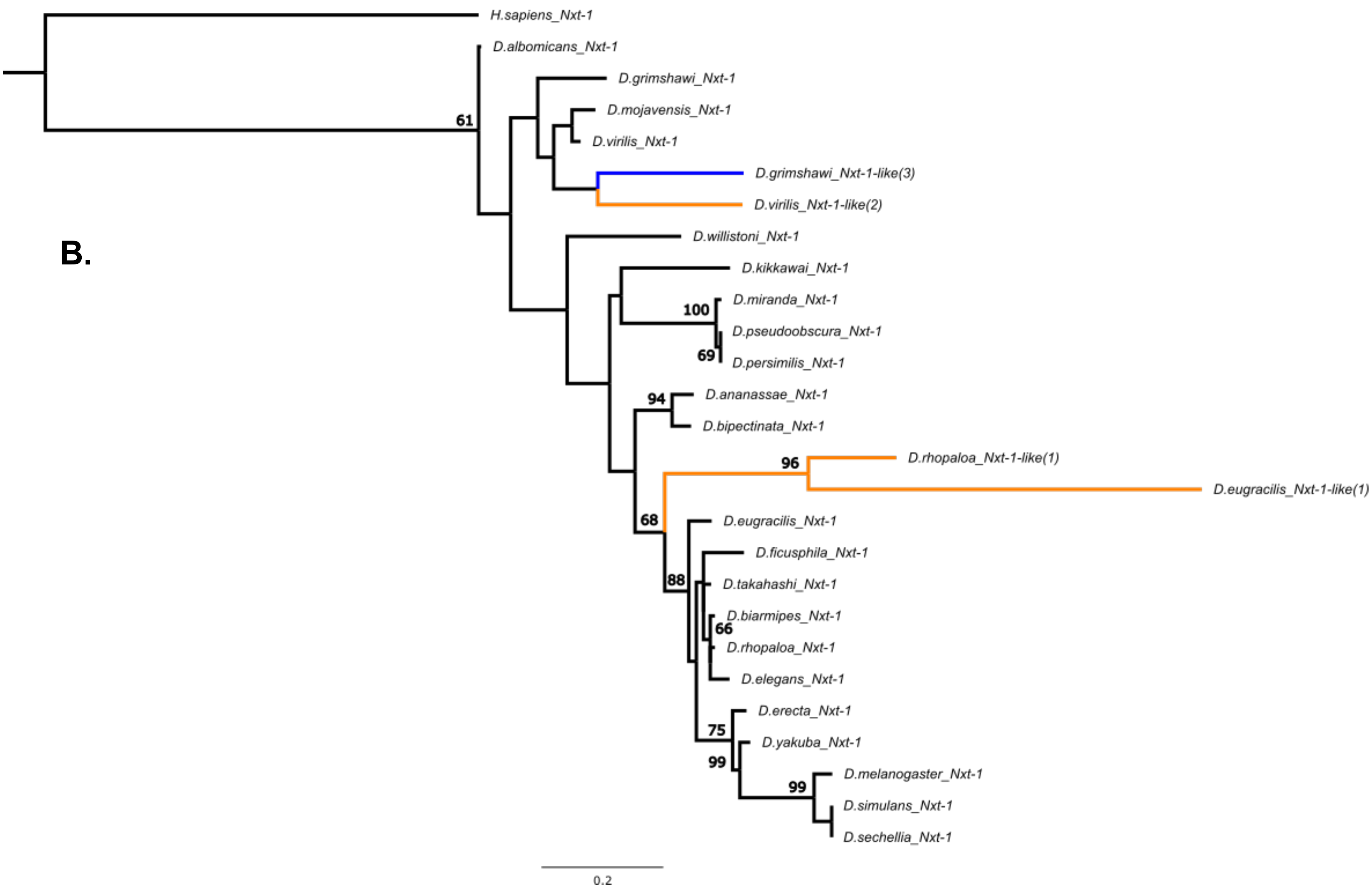

H.sapiens_Nxt-1
D.albomicans_Nxt-1
D.grimshawi_Nxt-1
D.mojavensis_Nxt-1
D.virilis_Nxt-1
D.grimshawi_Nxt-1-like(3)
D.virilis_Nxt-1-like(2)
D.willistoni_Nxt-1
D.kikkawai_Nxt-1
D.miranda_Nxt-1
D.pseudoobscura_Nxt-1
D.persimilis_Nxt-1
D.ananassae_Nxt-1
D.bipectinata_Nxt-1
D.rhopaloa_Nxt-1-like(1)
D.eugracilis_Nxt-1-like(1)
D.eugracilis_Nxt-1
D.ficusphila_Nxt-1
D.takahashi_Nxt-1
D.biarmipes_Nxt-1
D.rhopaloa_Nxt-1
D.elegans_Nxt-1
D.erecta_Nxt-1
D.yakuba_Nxt-1
D.melanogaster_Nxt-1
D.simulans_Nxt-1
D.sechellia_Nxt-1
61
100
69
94
96
68
88
66
75
99
99
0.2

C.

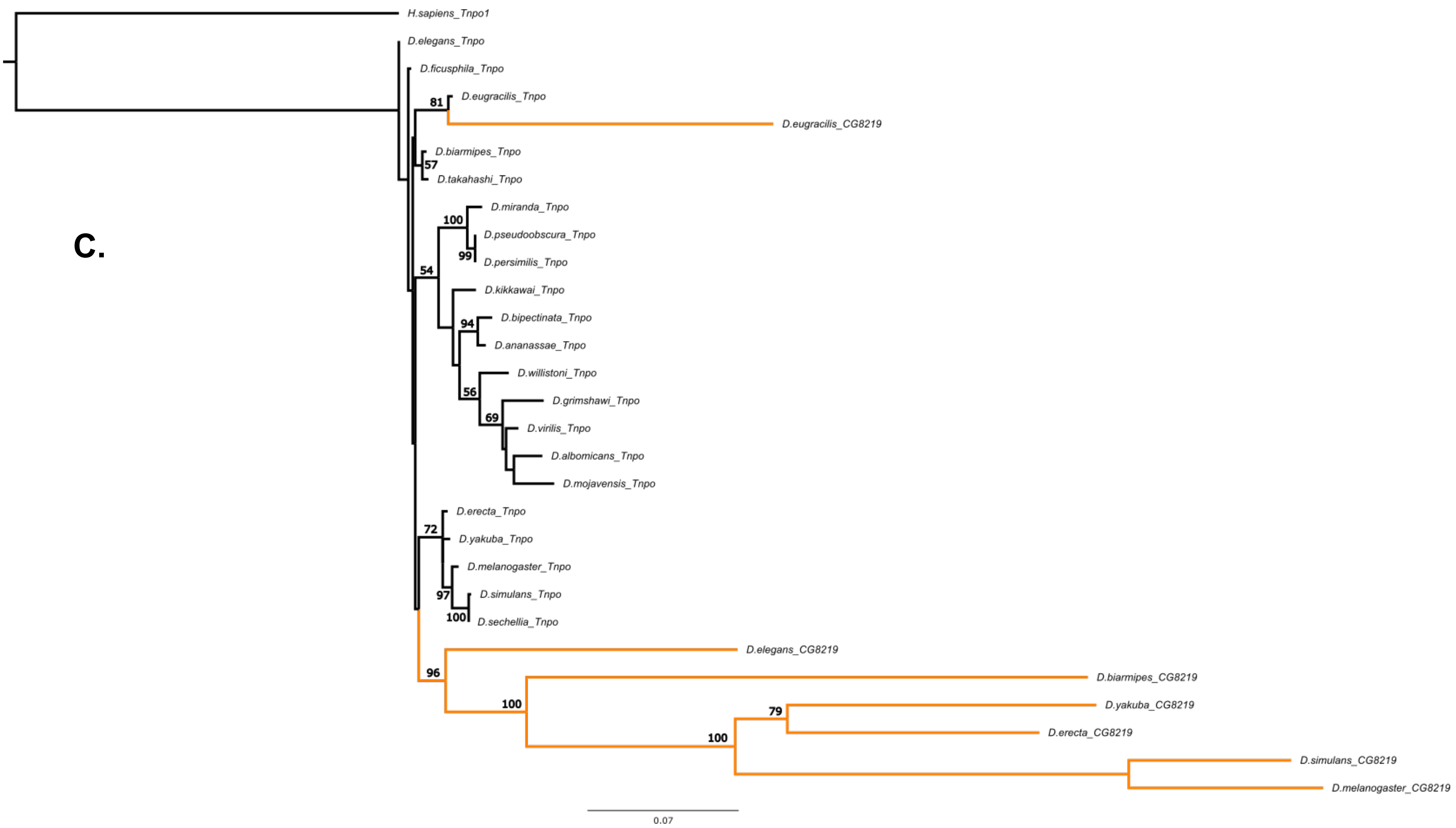
H.sapiens_Tnpo1
D.elegans_Tnpo
D.ficusphila_Tnpo
81
D.eugracilis_Tnpo
D.eugracilis_CG8219
D.biarmipes_Tnpo
57
D.takahashi_Tnpo
100
D.miranda_Tnpo
D.pseudoobscura_Tnpo
99
D.persimilis_Tnpo
54
D.kikkawai_Tnpo
94
D.bipectinata_Tnpo
D.ananassae_Tnpo
D.willistoni_Tnpo
56
D.grimshawi_Tnpo
69
D.virilis_Tnpo
D.albomicans_Tnpo
D.mojavensis_Tnpo
D.erecta_Tnpo
72
D.yakuba_Tnpo
D.melanogaster_Tnpo
97
D.simulans_Tnpo
100
D.sechellia_Tnpo
D.elegans_CG8219
96
D.biarmipes_CG8219
100
79
D.yakuba_CG8219
D.erecta_CG8219
100
D.simulans_CG8219
D.melanogaster_CG8219
0.07

**D.**

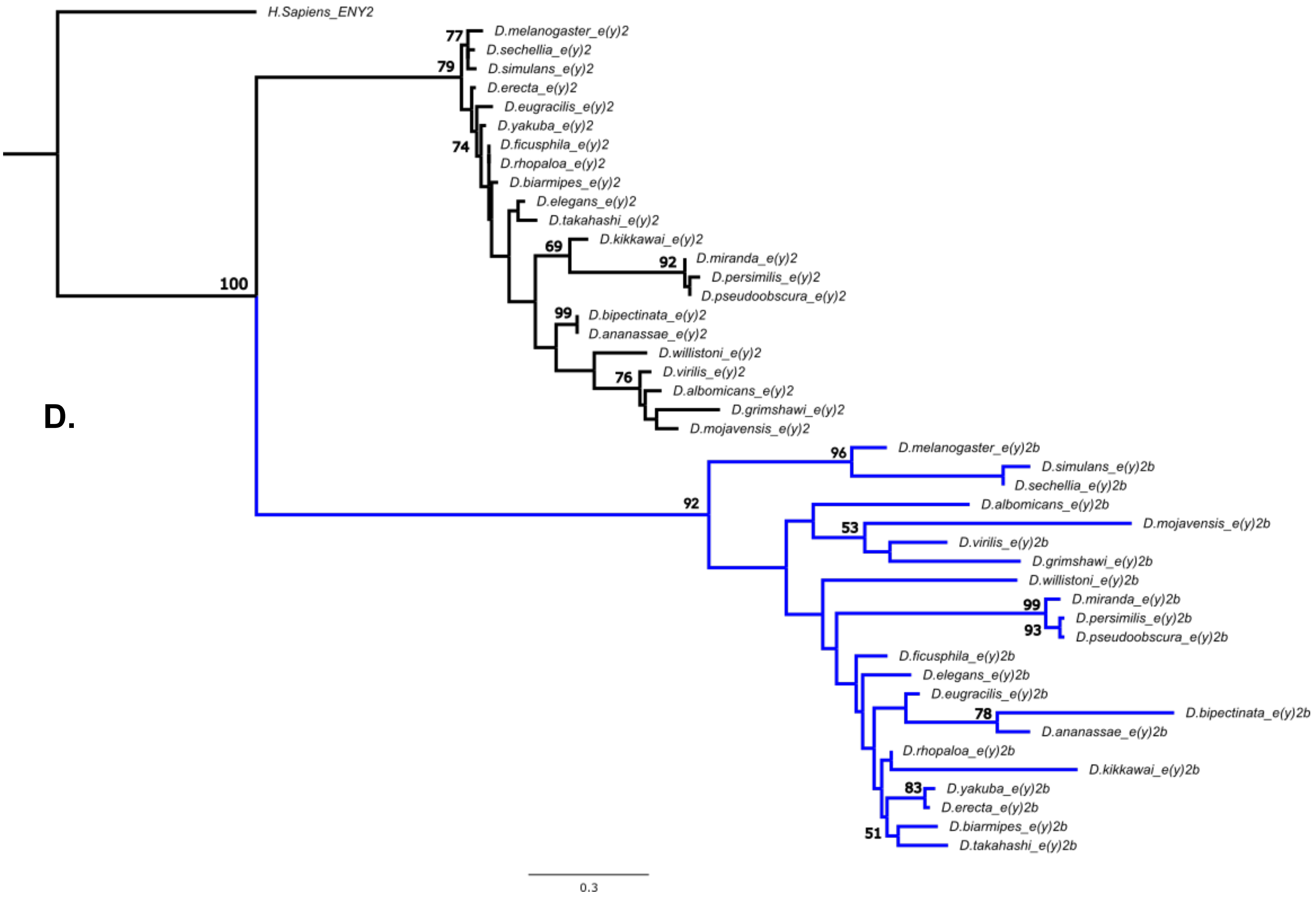
H.Sapiens_ENY2
D.melanogaster_e(y)2
D.sechellia_e(y)2
D.simulans_e(y)2
D.erecta_e(y)2
D.eugracilis_e(y)2
D.yakuba_e(y)2
D.ficusphila_e(y)2
D.rhopaloa_e(y)2
D.biarmipes_e(y)2
D.elegans_e(y)2
D.takahashi_e(y)2
D.kikkawai_e(y)2
D.miranda_e(y)2
D.persimilis_e(y)2
D.pseudoobscura_e(y)2
D.bipectinata_e(y)2
D.ananassae_e(y)2
D.willistoni_e(y)2
D.virilis_e(y)2
D.albomicans_e(y)2
D.grimshawi_e(y)2
D.mojavensis_e(y)2
D.melanogaster_e(y)2b
D.simulans_e(y)2b
D.sechellia_e(y)2b
D.albomicans_e(y)2b
D.mojavensis_e(y)2b
D.virilis_e(y)2b
D.grimshawi_e(y)2b
D.willistoni_e(y)2b
D.miranda_e(y)2b
D.persimilis_e(y)2b
D.pseudoobscura_e(y)2b
D.ficusphila_e(y)2b
D.elegans_e(y)2b
D.eugracilis_e(y)2b
D.bipectinata_e(y)2b
D.ananassae_e(y)2b
D.rhopaloa_e(y)2b
D.kikkawai_e(y)2b
D.yakuba_e(y)2b
D.erecta_e(y)2b
D.biarmipes_e(y)2b
D.takahashi_e(y)2b
77
79
74
69
92
99
76
100
96
92
53
99
93
78
83
51
0.3

**Supplmentary material 5.** Maximum-likelihood tree constructed using PhyML showing the phylogenetic relationships between parental and duplicates of *Nup93* (A), *Nxt-1* (B), *Tnpo* (C) and *e(y)2b* (D) based on amino acid sequences. DNA mediated duplication are shown in orange and RNA-mediated duplications are shown in blue. Bootstrap values refer to 100 trials on PhyML performed using geneious software.
